# Extracellular Granzyme K Modulates Angiogenesis by Regulating Soluble VEGFR1 Release From Endothelial Cells

**DOI:** 10.3389/fonc.2021.681967

**Published:** 2021-06-09

**Authors:** Shuang Li, Christian G. M. van Dijk, Jan Meeldijk, Helena M. Kok, Isabelle Blommestein, Annick L. F. Verbakel, Marit Kotte, Roel Broekhuizen, Miangela M. Laclé, Roel Goldschmeding, Caroline Cheng, Niels Bovenschen

**Affiliations:** ^1^ Department of Pathology, University Medical Center Utrecht, Utrecht, Netherlands; ^2^ Department of Nephrology and Hypertension, University Medical Center Utrecht, Utrecht, Netherlands; ^3^ Center for Translational Immunology, University Medical Center Utrecht, Utrecht, Netherlands

**Keywords:** colorectal cancer, angiogenesis, endothelium, soluble VEGFR1, granzyme K

## Abstract

Angiogenesis is crucial for normal development and homeostasis, but also plays a role in many diseases including cardiovascular diseases, autoimmune diseases, and cancer. Granzymes are serine proteases stored in the granules of cytotoxic cells, and have predominantly been studied for their pro-apoptotic role upon delivery in target cells. A growing body of evidence is emerging that granzymes also display extracellular functions, which largely remain unknown. In the present study, we show that extracellular granzyme K (GrK) inhibits angiogenesis and triggers endothelial cells to release soluble VEGFR1 (sVEGFR1), a decoy receptor that inhibits angiogenesis by sequestering VEGF-A. GrK does not cleave off membrane-bound VEGFR1 from the cell surface, does not release potential sVEGFR1 storage pools from endothelial cells, and does not trigger sVEGFR1 release *via* protease activating receptor-1 (PAR-1) activation. GrK induces *de novo* sVEGFR1 mRNA and protein expression and subsequent release of sVEGFR1 from endothelial cells. GrK protein is detectable in human colorectal tumor tissue and its levels positively correlate with sVEGFR1 protein levels and negatively correlate with T4 intratumoral angiogenesis and tumor size. In conclusion, extracellular GrK can inhibit angiogenesis *via* secretion of sVEGFR1 from endothelial cells, thereby sequestering VEGF-A and impairing VEGFR signaling. Our observation that GrK positively correlates with sVEGFR1 and negatively correlates with angiogenesis in colorectal cancer, suggest that the GrK-sVEGFR1-angiogenesis axis may be a valid target for development of novel anti-angiogenic therapies in cancer.

## Introduction

In angiogenesis, the vasculature is expanded by new branches sprouting from existing blood vessels (1). This process is crucial for human development and tissue repair, but also plays a key role in many diseases, including inflammatory diseases, cardiovascular diseases, and cancer (2). The dominant endothelial receptor in angiogenesis is vascular endothelial growth factor-receptor 2 (VEGFR2) (3). Its activation by VEGF-A leads to endothelial cell proliferation and migration, a starting point for capillary sprouting. VEGF-A can also bind the pro-angiogenic VEGF-receptor 1 (VEGFR1), a kinase-impaired receptor with higher affinity for VEGF-A as compared to VEGFR2. VEGFR1 also exists in a soluble form, a splice variant of the full-length receptor encoding only six of the seven extracellular immunoglobulin domains (4). Release of sVEGFR1 provides a negative feedback loop of angiogenesis by sequestering VEGF-A and thereby preventing it from binding to VEGFRs (5–8). For instance, soluble VEGFR1 (sVEGFR1) is overexpressed and has a prognostic value in colorectal cancer (9).

Granzymes together with the pore-forming protein perforin are stockpiled in granules of cytotoxic lymphocytes (*i.e.*, CD8+ T cells, natural killer (NK) cells, NKT cells, and γδ T cells) (10), which are released into the immunological synapse upon recognition of target cells. Granzymes are a family of structurally related serine proteases that are best known for their ability to induce apoptosis upon perforin-mediated delivery in tumor cells or virus-infected cells (11). There are five members in humans: granzyme A(GrA), GrB, GrH, GrK, and GrM (12). Despite similarities in structure and sequence, their substrate specificities overlap only partially (13, 14).

Elevated granzyme levels are also detected in the circulation in many diseases, including autoimmune diseases, atherosclerosis, pulmonary diseases, but also during infection or inflammation (15–17). Increasing evidence is emerging that granzymes exert noncytotoxic extracellular functions in inflammation, extracellular matrix degeneration, and wound healing (18–20). GrK is expressed by macrophages and plays a pathogenic role in inflammation, re-epithelialization, and matrix remodeling during wound healing (21). GrK can enhance TLR4-mediated proinflammatory cytokine response in monocytes and can directly trigger proinflammatory cytokines from fibroblasts and endothelial cells *via* cleavage and activation of protease activating receptor-1 (PAR-1) (22–24). The role of GrK in angiogenesis remains unknown.

In the present study, we demonstrate that extracellular GrK inhibits angiogenesis by triggering *de novo* synthesis and release of sVEGFR1 from endothelial cells. This leads to sequestering of VEGF-A and inhibition of VEGFR signaling and subsequent angiogenesis. GrK protein is detectable in human colorectal tumor tissue and its levels positively correlate with sVEGFR1 protein levels and negatively correlate with T4 intratumoral angiogenesis.

## Materials and Methods

### Granzymes

GrK and GrKSA were produced as described previously (25). Briefly, Human GrK and its catalytically inactive mutant GrKSA, in which a serine has been replaced with Ala, were produced in *Pichia pastoris* and purified using cation-exchange chromatography. After dialysis granzymes were further purified (to >98% homogeneity) by affinity-chromatography using Protein A/G Ultralink beads (Thermo Scientific) and the catalytic activity was confirmed using the synthetic chromogenic substrate Ac-Lys-pNA (Bachem) and cleavage of macromolecular substrates. The GrK concentrations used in our experiments are based on previous work that studied extracellular effects of granzymes (23, 24).

### Cell Culture

Pooled human umbilical vein endothelial cells (HUVECs) were cultured in EGM-2 (endothelial growth medium, consisting of EBM-2 (endothelial basal medium) supplemented with the EGM-2 bullit kit (Lonza). Cells were maintained under standard growth conditions (at 37°C, 5% CO_2_) and used for experiments between passage 3-5. All experiments were preceded by a 2-hour serum starvation in EBM-2 supplemented with 100 units/ml penicillin and 100 µg/ml streptomycin (pen/strep). Treatment of cells was performed in EBM-2 supplemented with 0.2% fetal calf serum (FCS) and pen/strep (Invitrogen). To determine the condition of the cells, a viability assay was performed using Cell Proliferation reagent WST-1 (Roche).

For biosynthesis inhibition, 18 µM cycloheximide (Sigma-Aldrich) were pre-incubated with cells for 2 hours before adding GrK or GrKSA. All were directly added to the wells for a 24 hour-treatment. Recombinant human VEGF165 protein was from R&D Systems. ATAP2 was from Santa Cruz Biotechnology.

### Angiogenic 3-Dimensional Co-Culture Assay

HUVEC (Lonza) cells were transfected with lentiviral GFP and human brain derived pericytes (Sciencell) were transfected with lentiviral Red Fluorescent Protein (dsRED), according to the manufacturer’s instructions (Dharmacon). HUVEC GFP were transfected with siVEGFR1 (siFlt1) and a non-targeting siRNA referred as siSHAM (ON-TARGETplus SMARTpool, Dharmacon) using Dharmafect 1 (Dharmacon). HUVEC GFP and pericytes dsRED were co-cultured at a 5:1 ratio in co-culture medium containing with EBM-2 basal medium, 2% FCS (Invitrogen), ascorbic acid and FGF from the EGM-2 bullit kit (Lonza). Then, 0.2 µg/ml of feline/human/rhesus macaque CXCL12/SDF1α, human SCF/c-kit ligand and human IL-3 (all R&D systems) were added. Subsequently, bovine collagen type 1 (Gibco) was added to the cells (final concentration of 2.2 mg/ml, pH7.4) in a 96 wells flat bottom plate. Polymerization of the protein gel was allowed for 1 hour at 37°C, 5% CO_2_. The gel was then overlaid with co-culture medium. Second day, different concentrations of GrK or GrKSA solutions were added to the gel. After 3 days, images were taken using an Olympus IX51 fluorescence microscope with CellSenseDimension software. Images were processed (inverted, image size and improved contrast) using Adobe Photoshop and analyzed using AngioSys (Cellworks).

### Luminex Assay

HUVEC cells were pelleted by centrifugation at 2000 rpm for 5 min before harvesting the conditioned medium. sVEGFR1 levels were measured in a Luminex assay, using the human anti-VEGFR1 antibody (clone 49560, R&D Systems). Measurements were performed using an in-house developed and validated (ISO9001 certified) multiplex immunoassay based on Luminex xMAP technology (Luminex, Austin TX USA) as described (26).

### Flow Cytometry

Cells (1 x 10^5^) were seeded in 12-well plates. After treatment, cells were washed in phosphate buffered saline (PBS) containing 1% bovine serum albumin (BSA), detached from the plate using Accutase and transferred to FACS tubes. 4% formalin was used for fixation, followed by washing and cells were incubated with primary antibody, Rabbit monoclonal anti-VEGFR1 antibody (clone Y103, Abcam, 1:100) for 30 min on ice. After incubation of the secondary antibody, Alexa Flour 488-conjugated goat anti-rabbit IgG (Thermo Fisher), cells were resuspended in 200 µl PBS/1% BSA and measured on a BD FACS Canto II flow cytometer (BD Bioscience). Data was analyzed with BD FACSDiva software.

### RT-PCR

Cells were treated with 500 nM GrK or 500 nM GrKSA. After 16 hours, 1 ml of TRIzol Reagent (Ambion) was added to homogenize the sample. After 5 minutes at room temperature, the RNA was extracted using chloroform. The RNA-phase was precipitated with isopropanol, pelleted by centrifugation at 10,000 rpm for 15 minutes, washed with 70% ethanol and dissolved in water. Subsequently, an equal amount of RNA was used to make cDNA. RNA expression levels were measured in a RT-qPCR reaction, using SYBR Green Dye (Applied Biosystems). All measurements were performed in duplicate on a ViiA 7 Real-Time PCR System (Applied Biosystems). Relative quantification results were obtained using the ΔΔCt method and expression values were normalized to ubiquitin C (UBC). Primers used in 5’ to 3’ direction: sVEGFR1 forward:AGAGGTGAGCACTGCAACAA, sVEGFR1 reverse: TCTCCTCCGAGCCTGAAAGT, UBC forward: AGTAGTCCCTTCTCGGCGAT and UBC reverse: GCATTGTCAAGTGACGATCACAG.

### Colorectal Cancer Tissue

Tumor and healthy specimen pairs (n=71) were obtained from colorectal cancer patients treated between 2009-2014 at the University Medical Center Utrecht (Utrecht, The Netherlands). The study specimens were distributed by the Biobank of the Department of Pathology, with the permission of the institutional medical review board (number 19/098). Patient characteristics are shown in **Table 1**.

**Table 1:** Protein levels of sVEGFR1 and GrK in colorectal cancer.

			sVEGFR1			GrK		
			Median/range (ng/mg)		P value	Median/range (ng/mg)		P value
			Control	Tumor		Control	Tumor	
	variables (n=71)	N/Percentage (%)	2.859/9.551	4.677/14.49	***<0.0001	0.08648/1.660	0.1309/1.007	*0.0146
Age - yr	≤60	14/19.7	1.945/5.680	3.905/11.42	**0.0023	0.06/1.050	0.145/0.4500	0.2373
	>60	57/80.3	2.99/9.460	4.77/13.93	***<0.0001	0.09/1.660	0.12/1.010	*0.0404
Gender	Male	38/53.5	2.32/9.460	5.14/11.42	***<0.0001	0.08/1.650	0.15/0.5400	*0.0103
	Female	33/46.5	3.04/7.820	4.48/13.52	**0.0061	0.09/1.050	0.1/1.010	0.352
Location	Coecum	14/19.7	3.57/7.150	4.555/9.840	0.1937	0.095/0.3300	0.085/0.3700	0.5313
	Sigmoideum	30/42.3	1.815/6.580	5.36/14.48	***<0.0001	0.065/0.5800	0.145/1.010	*0.0245
	Ascendens	10/14.1	3.23/8.820	3.96/10.02	0.1309	0.09/1.650	0.29/0.7500	0.084
	Flexura hepatica	3/4.2	4.36/1.990	4.57/1.560	>0.9999	0.0/0.03000	0.21/0.1300	0.25
	Lienalis	2/2.8	1.41/0.3800	3.785/0.5300	0.3333	0.14/0.1400	0.17/0.1800	0.6667
	Transversum	9/12.7	3.76/5.800	4.96/6.800	0.25	0.09/1.010	0.06/0.4300	0.1875
	Descendens	3/4.2	2.93/5.540	3.34/5.630	0.5	0.28/0.2000	0.1/0.6600	>0.9999
Tumor stage	T2	13/18.3	2.43/8.940	4.8/10.87	**0.0024	0.045/1.650	0.11/0.7800	0.0598
	T3	44/62.0	2.585/7.790	4.655/14.48	***<0.0001	0.085/1.050	0.165/1.010	*0.0144
	T4	14/19.7	3.049/7.038	4.272/9.913	0.058	0.1086/0.3263	0.0804/0.2634	0.2676

*P < 0.05,**P < 0.01, ***P < 0.001 (indicated in bold); statistics of comparison analysed using Wilcoxon matched-pairs signed rank test.

To extract proteins, tissue samples (10 slices, 20 μm) were collected in tubes containing Precellys 1,4 mm zirconium oxide beads (Bertin Technologies). Subsequenty, 200 μl PBS (pH 7.4) with 1% Tween-20 and Complete EDTA-free protease inhibitor cocktail were added. Tissues were homogenized using a Precellys 24 (Bertin Technologies). After centrifugation the supernatants were stored at 4°C. Total protein concentration was determined using a BCA Assay kit (Thermo Fisher Scientific).

### Enzyme Linked Immunosorbent Assay (ELISA)

The concentration of sVEGFR1 in colorectal tissue extracts was determined by a human VEGFR1 ELISA kit (Thermo Fisher Scientific) according to the manufacturer’s protocol. The lower detection limit of sVEGFR1 was 0.16 ng/mL. Samples were diluted 1:16.7. The concentration of GrK in colorectal tissue extracts was measured using a human GrK ELISA kit (Aviva Systems Biology) according to the manufacturer’s protocol. The lower detection limit of GrK was 0.03 ng/mL. Samples were diluted 1:4.

### Immunohistochemistry (IHC)

Hematoxylin-Eosin (HE) staining and IHC were carried out on 4 μm thick formalin-fixed, paraffin-embedded sections. HE staining was performed by automatic stainers (Leica Biosystems). Immunohistochemistry for CD31 (DAKO, clone JC/70A, 1:100) was performed using an automated immunostainer (Benchmark Ultra, Ventana, Roche). Proteins were visualized using the OptiView DAB IHC Detection Kit (Roche).Whole tissue slides were scanned at magnification ×40 (NanoZoomer). Five 1.5 mm^2^ areas within the tumor were selected randomly for evaluation. The intensity scoring for CD31 staining was determined by ImageJ software with the plugin IHC profiler. The intensity of the staining was scored as mean of positive area.

### Statistical Analysis

Data using cell cultures were analyzed using an unpaired t-test when comparing 2 groups or a one-way ANOVA when comparing multiple groups. Data from patient samples were analyzed using Wilcoxon matched-pairs signed rank test. The correlation between two factors was evaluated using the Spearman’s correlation. All statistical evaluations were carried out using GraphPad Prism software. Results were considered statistically significant when *p* < 0.05.

## Results

### Extracellular GrK Inhibits Angiogenesis *In Vitro*

**Figure 1:**
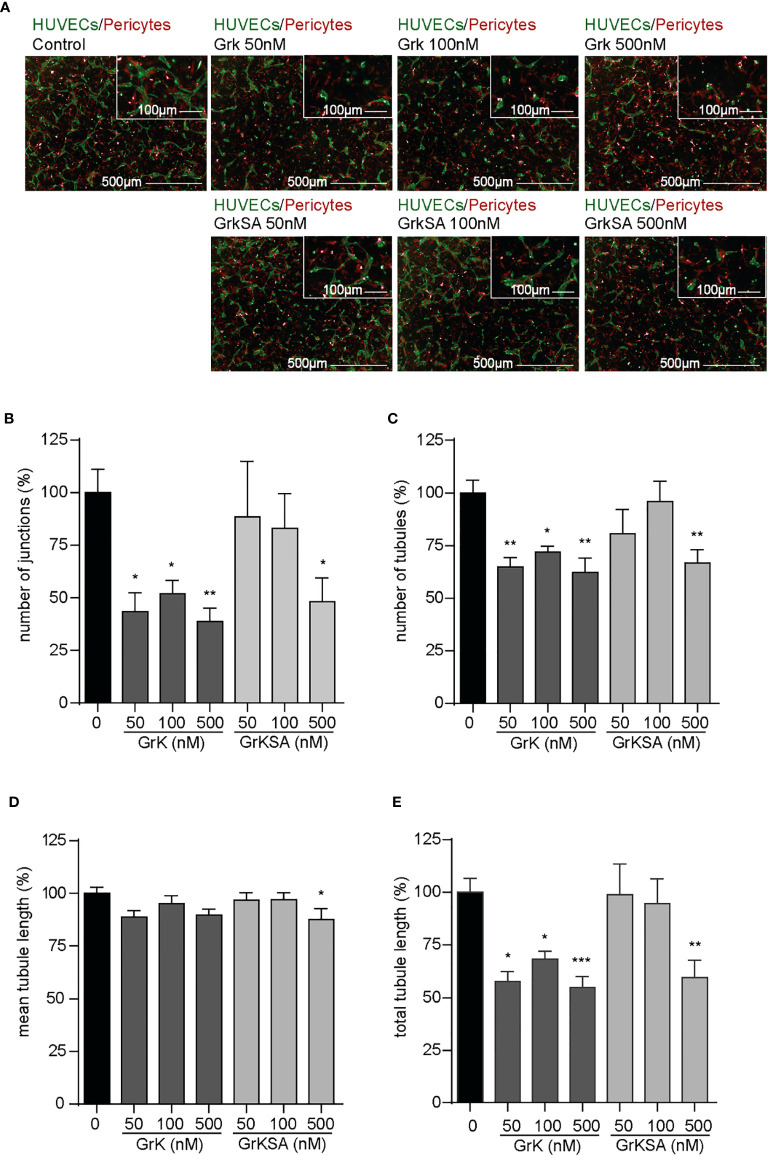
GrK inhibits angiogenesis *in vitro*. A co-culture of pericytes and endothelial cells in a 3D collagen gel was treated with a concentration series of GrK and GrKSA. **(A)** Representative fluorescent microscope images of GFP-labeled HUVECs (green) in co-culture with dsRED-labeled pericytes (red) in a 3D collagen matrix during vascular formation. Shown are the results at day 3 of an untreated control and after stimulation with different concentrations of GrK or GrKSA. Scale bar =500µm. Right corner corresponding to the inset, scale bar=100µm. Angiogenesis was described *via* **(B)** number of junctions, **(C)** number of tubules, **(D)** mean tubule length and **(E)** total tubule length. For all parameters, relative values compared to mock concentration (100%) are shown. Results are depicted as mean ± SEM, n≥10. *P < 0.05; **P < 0.01; ***P < 0.001, compared to control.

To study the role of extracellular GrK in angiogenesis, we used an angiogenic 3D co-culture assay with endothelial cells and pericytes. Representative images thereof are shown (**Figure 1A**). Treatment with different concentrations of GrK significantly decreased the number of junctions (**Figure 1B**), the number of tubules (**Figure 1C**), and the total tubule length (**Figure 1E**). The catalytically inactive GrKSA also reduced these parameters, although markedly less efficient as compared to GrK. In all cases, mean tubule length remained unaffected (**Figure 1D**). These data indicate that extracellular GrK inhibits angiogenesis *in vitro*.

### GrK Triggers the Release of sVEGFR1 From Endothelial Cells

**Figure 2:**
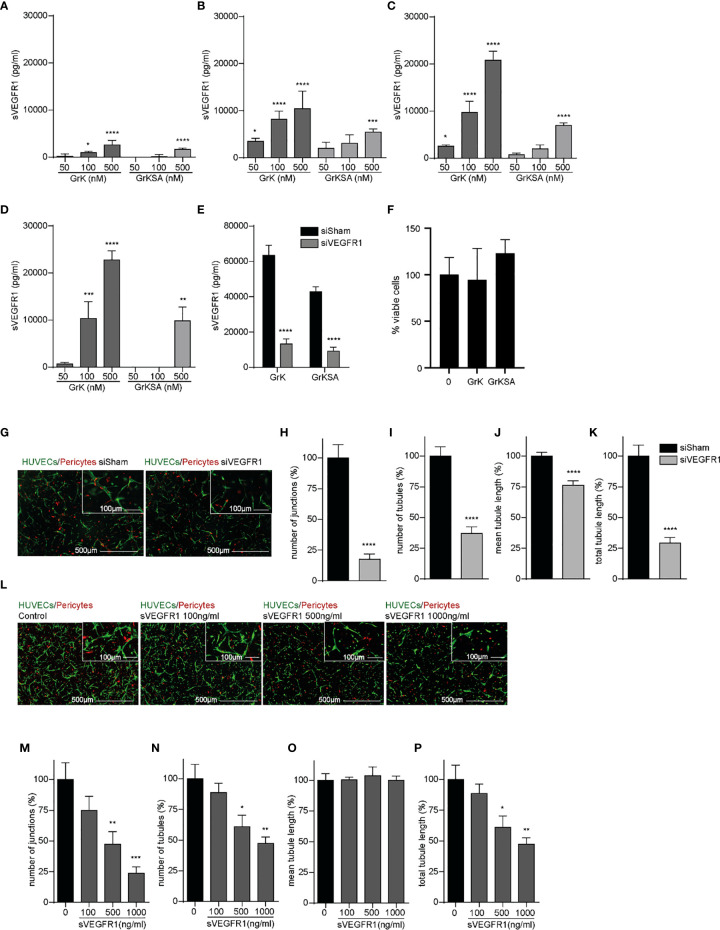
GrK induces sVEGFR1 release from endothelial cells. HUVECs were treated with different concentrations of GrK or GrKSA. The conditioned medium was harvested and the level of sVEGFR1 was measured by Luminex. A time and concentration dependent increase of sVEGFR1 levels was observed after GrK(SA) treatment for **(A)** 1h, **(B)** 6h, **(C)** 24h, and **(D)** 48h. Data were corrected for background signal, n=4. **(E)** HUVEC GFP cells were transfected with siVEGFR1 (siFlt1) and a non-targeting siRNA (siSham). After incubation with 500 nM GrK(SA), inhibition of VEGFR1 shows significant lower sVEGFR1 levels. n=12. **(F)** HUVEC GFP cells were incubated with 500nM of GrK(SA) and detected by cell proliferation reagent WST-1. n=4. **(G–K)** The effect of VEGFR1 inhibition in an angiogenic 3D co-culture assay was investigated for each parameter. Representative fluorescent microscope images of GFP-labeled HUVECs(green) in co-culture with dsRED-labeled pericytes (red) in a 3D collagen matrix during vascular formation. Shown are the results of siSHAM and siVEGFR1. Scale bar =500µm. Right corner corresponding to the inset, scale bar=100µm. n≥15. **(L–P)** Effect of sVEGFR1 release was investigated by directly adding sVEGFR1 to the angiogenic co-culture assay. Representative fluorescent microscope images of GFP-labeled HUVECs (green) in co-culture with dsRED-labeled pericytes (red) in a 3D collagen matrix during vascular formation. Shown are the results of an untreated control and after stimulation with different concentrations of sVEGFR1. Scale bar=500µm. Right corner corresponding to the inset, scale bar=100µm.n≥6. Results are depicted as mean ± SEM. *P < 0.05; **P < 0.01; ***P < 0.001; ****P < 0.0001, compared to control.

Since sVEGFR1 is an important negative modulator of angiogenesis by sequestering VEGF-A and VEGFR2 activation (27), we investigated the effect of extracellular GrK on sVEGFR1 release in the medium of endothelial cells. GrK dose-dependently induced sVEGFR1 levels in cell culture media after 1 hour (**Figure 2A**), 6 hours (**Figure 2B**), 24 hours (**Figure 2C**) and 48 hours (**Figure 2D**) of incubation. At all timepoints, the highest concentration of GrKSA (500 nM) also induced sVEGFR1 accumulation in the media, but to a lesser extent. To confirm specificity of sVEGFR1 detection by luminex, siRNA for (s)VEGFR1 was performed in combination with administration of GrK and GrKSA. As expected, knockdown of (s)VEGFR1 dramatically decreased sVEGFR1 release by GrK as measured by luminex (**Figure 2E**). Both extracellular GrK and GrKSA did not influence cell viability (**Figure 2F**). Knockdown of VEGFR1 or adding purified sVEGFR1 in our angiogenic co-culture system with endothelial cells, pericytes and matrix proteins reduced angiogenesis, confirming the importance of membrane-bound and sVEGFR1 (**Figures 2G–K** and **2L–P**, respectively) (8, 28). Taken together, we conclude that GrK, and to lesser extent GrKSA, induces the release of sVEGFR1 from endothelial cells.

### GrK Does Not Cleave Membrane-Bound VEGFR1

**Figure 3:**
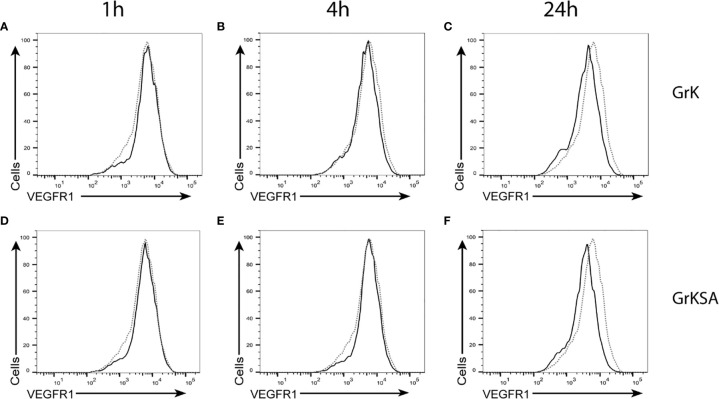
GrK does not cleave membrane-bound VEGFR1. HUVECs were treated with 500nM GrK or GrKSA and the amount of membrane-bound VEGFR1 was measured by flow cytometry. Incubation for **(A)** 1h, **(B)** 4h, **(C)** 24h of GrK and **(D)** 1h, **(E)** 4h, **(F)** 24h of GrKSA is shown. X-axes show anti-VEGFR1 antibody (logarithm of fluorescence), Y-axes depict the cell count. The signal for untreated cells is depicted in dotted line. The signal for GrK- or GrKSA-treated cells is depicted in solid black line. Figures are representative experiments (n=3).

To investigate whether GrK induces sVEGFR1 shedding by cleaving off the membrane-bound receptor, endothelial cells were treated with GrK or GrKSA and the amount of membrane-bound VEGFR1 was measured by flow cytometry. No significant shifts in VEGFR1 positive stainings were observed after GrK (**Figures 3A–C**) or GrKSA (**Figures 3D–F**) treatment compared to mock, following 1, 6, and 24 hours of incubation. Thus, GrK does not cleave VEGFR1 from the endothelial cell membrane.

### GrK Induces sVEGFR1 Release From Endothelial Cells Independently of PAR-1

**Figure 4:**
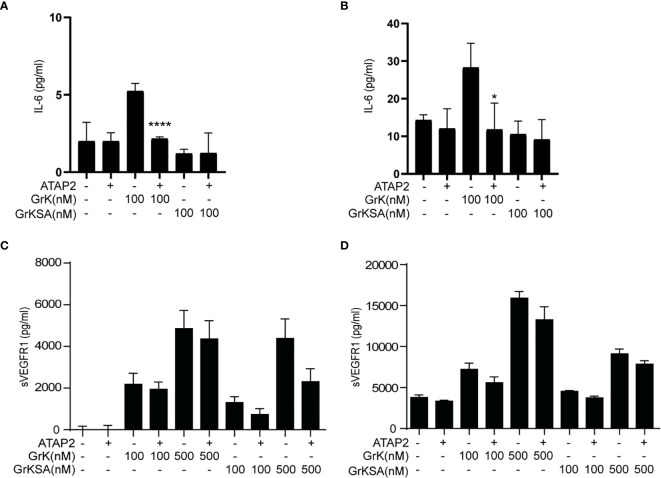
sVEGFR1 release is not PAR-1-dependent. HUVECs were treated with different concentrations of GrK or GrKSA with or without PAR-1 inhibitor ATAP2. IL-6 was used as a positive control for PAR-1 dependent cytokine release after **(A)** 6h and **(B)** 24h of incubation. After **(C)** 6h and **(D)** 24h of incubation with GrK(SA) no PAR-1 dependent release of sVEGFR1 was seen. Results are depicted as mean ± SEM,n=3. *P < 0.05; ****P <0.0001, compared to its condition without ATAP2.

It has been established that GrK can mediate proinflammatory cytokine (*e.g.*, IL-6) release from endothelial cells *via* cleavage and activation of PAR-1, which is the only known membrane receptor that GrK can activate to initiate intracellular signal transduction (22). Therefore, we investigated whether GrK-dependent sVEGFR1 release depends on PAR-1. IL-6 production was found after 6 (**Figure 4A**) and 24 hours (**Figure 4B**) following incubation with GrK, but not GrKSA. As expected (22), IL-6 production was directly inhibited by PAR-1 inhibitor ATAP2. GrK, and to lesser extent GrKSA induced sVEGFR1 release after 6 (**Figure 4C**) and 24 hours (**Figure 4D**). This effect was not dependent on PAR-1 since co-incubation with ATAP2 did not affect sVEGFR1 levels. These data indicate that GrK induces sVEGFR1 release from endothelial cells independent of PAR-1.

### GrK Induces VEGFR1 Protein and mRNA Levels

**Figure 5:**
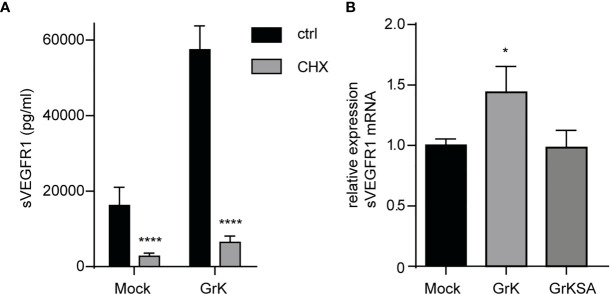
GrK potentiates *de novo* VEGFR1 mRNA and protein expression. **(A)** HUVECs were pre-treated with cycloheximide (CHX) to inhibit protein translation and subsequently treated for 24 hours with 500nM GrK or GrKSA. sVEGFR1 levels in the medium were measured by Luminex. **(B)** HUVECs were treated for 16 hours with 500nM GrK or 500nM GrKSA and sVEGFR1 mRNA expression was measured in a RT-qPCR assay. Results are depicted as mean ± SEM, n≥3. *P < 0.05; ****P < 0.0001, compared to Mock value.

Next, we investigated whether GrK triggers *de novo* synthesis of the soluble splice variant of VEGFR1, or that it induces release from storage vesicles within endothelial cells. Endothelial cells were pre-treated with cycloheximide, an inhibitor of translation (29), and subsequently treated for 24 hours with GrK or GrKSA. Cycloheximide treatment dramatically reduced GrK-induced sVEGFR1 release to levels below baseline (**Figure 5A**). These data suggest that the release of sVEGFR1 induced by GrK depends on *de novo* protein synthesis in the endothelial cell. To establish whether GrK triggers *de novo* synthesis of sVEGFR1 mRNA transcription, we performed RT-qPCR reaction with a primer pair specific for the soluble splice variant of VEGFR1. A 16-hour GrK treatment increased sVEGFR1 mRNA expression, whereas GrKSA treatment showed baseline expression levels (**Figure 5B**). These data show that extracellular GrK induces *de novo* up-regulation of sVEGFR1 mRNA and protein.

### GrK Protein Is Detectable and Positively Correlates With sVEGFR1 in Human Colorectal Tumor Tissue

**Figure 6:**
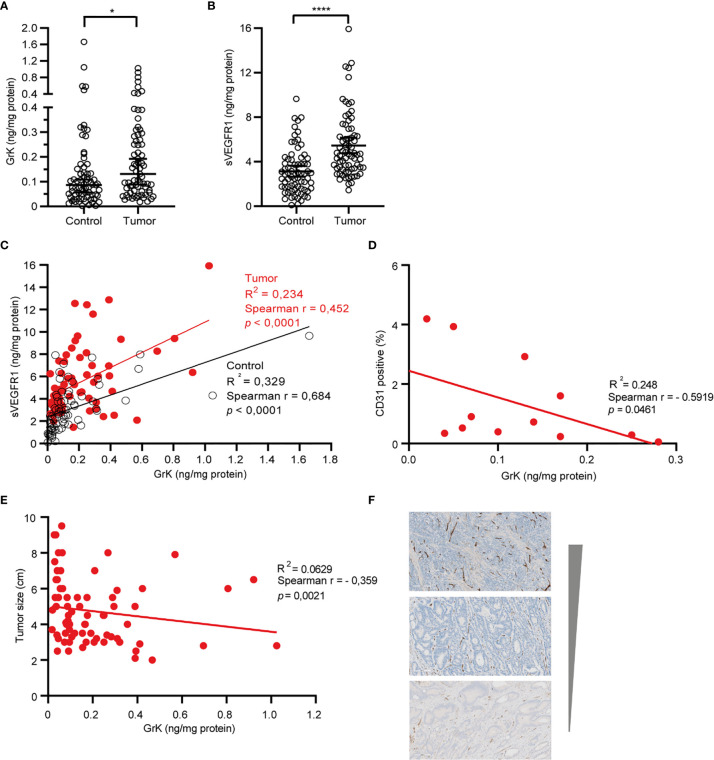
GrK and sVEGFR1 protein levels correlate in colorectal cancer tissue **(A)** Comparison of the concentration of sVEGFR1 in colorectal cancer and normal control (Wilcoxon signed rank test). Data were corrected for total protein levels. **(B)** Comparison of the concentration of GrK in colorectal cancer and normal control (Wilcoxon signed rank test). Data were corrected for total protein levels. **(C)** Correlation between sVEGFR1 and GrK concentrations in colorectal cancer and in normal control (Spearman’s rank correlation). **(D)** correlation between endothelial cells by CD31 and GrK in T4 colorectal cancer (Spearman’s rank correlation). **(E)** Correlation between GrK and Tumor size in colorectal cancer (Spearman’s rank correlation). **(F)** CD31 IHC staining of colorectal tumors. Three representative images are shown with high, intermediate and low amounts of angiogenesis Results are depicted as median and 95% CI. *P < 0.05; ****P < 0.0001.

If GrK were to play a role in the regulation of sVEGFR1 levels *in vivo*, one would expect that GrK protein levels are detectable in the tumor microenvironment and positively correlate with sVEGFR1. We initially chose to measure GrK in colorectal cancer tissue specimens, since sVEGFR1 protein levels are detectable in this type of cancer (9). To this end, we measured GrK and sVEGFR1 protein levels in human colorectal tumor tissue (n=71) and paired healthy control colon mucosa from the same patient. GrK protein was detected and the levels were increased approximately 1.6-fold in tumor tissue as compared to healthy mucosa (**Figure 6A**). Increased GrK protein levels associated with patient age (> 60, *P*=0.04), gender (male, *P*=0.01), tumor malignancy grade III (*P*=0.01), and sigmoid with location in the sigmoid (*P*=0.0245) (**Table 1**). As expected (9), sVEGFR1 protein levels were increased about 2-fold in tumor tissue as compared to healthy mucosa (**Figure 6B**). We found a significant correlation between GrK and sVEGFR1 protein levels both in colorectal cancer tissue and in normal mucosa tissue (**Figure 6C** and **Table 2**). Next, we examined angiogenesis of colorectal tumors by counting CD31+ endothelial cells (**Figure 6F** partially). GrK protein levels negatively correlated to the number of CD31+ endothelial cells in stage IV colorectal cancer (**Figure 6D** and **Table 2**). Finally, GrK protein levels negatively correlated with tumor size (**Figure 6E** and **Table 2**). Collectively, these data show that GrK protein is detectable in human colorectal tumor tissue, and that its levels positively correlate with sVEGFR1 protein levels and negatively correlate with T4 intratumoral angiogenesis and tumor size.

**Table 2:** Correlation between sVEGFR1, Tumor size, CD31 and GrK.

			Correlation sVEGFR1 *vs* GrK		Correlation Tumor Size *vs* Tumor GrK		Correlation Tumor GrK *vs* CD31	
			control	tumor	mean ± SD (cm)	Correlation	mean ± SD (%)	Correlation
			r/P value	r/P value	Tumor size	r/P value	CD31^+^ cells^#^	r/P value
	variables (n=71)	N/Percentage (%)	0.684/***<0.0001	0.452/***<0.0001	4.737 ± 1.829	-0.3593/**0.0021	5.635 ± 4.006	-0.005/0.967
Age - yr	≤60	14/19.7	0.788/**0.001	0.447/0.109	4.457 ± 1.894	-0.3705/0.191	6.182 ± 4.922	-0.2347/0.4600
	>60	57/80.3	0.654/***<0.0001	0.442/**0.001	4.730 ± 1.836	-0.3778/**0.0038	6.308 ± 3.639	0.08071/0.5618
Gender	Male	38/53.5	0.711/***<0.0001	0.369/*0.023	4.416 ± 1.701	-0.131/0.4343	6.352 ± 3.954	0.1093/0.5319
	Female	33/46.5	0.614/***<0.0001	0.495/**0.003	5.088 ± 1.947	-0.4795/**0.0048	5.861 ± 3.532	0.06432/0.7310
Location	Coecum	14/19.7	0.462/0.097	0.396/0.161	4.743 ± 1.781	-0.4492/0.1079	6.564 ± 3.344	0.01322/0.9659
	Sigmoideum	30/42.3	0.777/***<0.0001	0.585/**0.001	5.830 ± 7.212	-0.01606/0.9329	6.354 ± 4.606	-0.1492/0.4485
	Ascendens	10/14.1	0.879/**0.002	-0.389/0.266	5.010 ± 2.346	-0.1098/0.7617	6.856 ± 3.846	0.2929/0.4422
	Flexura hepatica	3/4.2	0.5/1	1/0.333	5.067 ± 1.901	-0.5/>0.9999	4.345 ± 2.270	ND/ND
	Lienalis	2/2.8	ND/ND	ND/ND	4.450 ± 1.485	ND/ND	4.701 ± 4.101	ND/ND
	Transversum	9/12.7	0.711/*0.037	0.819/*0.01	5.167 ± 2.337	-0.7416/*0.0275	4.969 ± 1.492	0.5636/0.1174
	Descendens	3/4.2	0.5/1	1/0.333	3.400 ± 0.520	-0.866/0.6667	4.540	ND/ND
Tumor stage	T2	13/18.3	0.863/***<0.0001	0.863/***<0.0001	3.500 ± 1.050	-0.37/0.2114	7.635 ± 3.65	0.1553/0.6471
	T3	44/62.0	0.652/***<0.0001	0.232/0.129	4.800 ± 1.834	-0.3313/*0.028	5.973 ± 3.611	0.1157/0.4601
	T4	14/19.7	0.433/0.122	0.873/***<0.0001	7.671 ± 0.990	-0.3028/0.289	5.416 ± 4.637	-0.5919/*0.0461

*P < 0.05,**P < 0.01, ***P < 0.001 (indicated in bold); ND, not determined (due to low n); statistics of analysed using Spearman’s correlation. ^#^n = 66.

## Discussion

In the present study, we have demonstrated that extracellular GrK can inhibit angiogenesis and that GrK can trigger endothelial cells to release sVEGFR1, a decoy receptor that inhibits angiogenesis by sequestering VEGF-A. GrK induces *de novo* sVEGFR1 mRNA and protein expression and subsequent release of sVEGFR1 from endothelial cells. GrK protein is detectable in human colorectal tumor tissue and its levels positively correlate with sVEGFR1 protein levels and negatively correlate with T4 intratumoral angiogenesis. Thus, extracellular GrK can inhibit angiogenesis *via* secretion of sVEGFR1 from endothelial cells, thereby sequestering VEGF-A and impairing VEGFR signaling.

The molecular mechanism by which GrK triggers sVEGFR1 release from endothelial cells remains unknown. We ruled out the possibility that GrK directly cleaves membrane-bound VEGFR1 from the cell surface (**Figure 3**). We also excluded that sVEGFR1 release relies on GrK-mediated cleavage and activation of the PAR-1 receptor, which is a known GrK target (22, 23). In this context, we cannot exclude that GrK activates other PAR family members. Moreover, GrK does not release existing sVEGFR1 storage pools from endothelial cells (**Figure 5**). Interestingly, we show that the catalytically inactive mutant of GrK, GrKSA, also can trigger sVEGFR1 release from endothelial cells although to a lesser extent (**Figures 2A–D**). This points to at least two mechanisms that are dependent and independent of GrK catalytic activity, respectively. The former might be most important, since catalytically active GrK was markedly more efficient in sVEGFR1 release as compared with the enzymatically inactive mutant GrKSA. Further study is required to elucidate the molecular mechanisms by which GrK triggers sVEGFR1 release from endothelial cells.

It is becoming more recognized that granzymes not solely trigger cell death in target cells, but that they also can exert multiple other functions both inside cells and in the extracellular milieu (17, 30). Once in the extracellular matrix, granzymes can contribute to inflammation, vascular dysfunction, vascular permeability, reduced cell adhesion, release of matrix-bound growth factors, extracellular matrix remodeling, re-epithelialization, and remodeling during wound healing (30). The importance of extracellular granzymes, however, remains poorly understood. Several studies have demonstrated physiological relevance of noncytotoxic functions of extracellular GrB, including tissue remodeling and angiogenesis (30–32). Extracellular GrB cleaves fibronectin, resulting in a reduction of endothelial cell adhesion to fibronectin as well as reduced endothelial cell migration and tubule formation (33). It has been demonstrated that extracellular GrK influences inflammation (22–24) and affects wound healing by impeding epithelialization (21). We now show that extracellular GrK can modulate angiogenesis by regulating soluble VEGFR1 release from endothelial cells. The *in vivo* relevance thereof remains to be established, but may be involved in vascular diseases, wound healing and/or cancer. For that matter, we demonstrated that both sVEGFR1 and GrK are detectable in both tumor and control tissues of colorectal cancer patients (**Figures 6A, B**). Remarkably, GrK protein levels correlated positively with sVEGFR1 protein levels and correlated negatively with stage IVintratumoral angiogenesis and tumor size (**Table 2** and **Figure 6D**). In this context, it should be mentioned that the location and source of GrK in colorectal tissue remains unknown, since we have employed tissue lysates to measure GrK protein levels (**Tables 1**, **2**). GrK may exist extracellularly in the tumor tissue microenvironment, but could also be present inside (the granules) of cytotoxic cells or other cells including macrophages, *i.e.*, the cells that can produce GrK (34). Further study is required to distinguish between these possibilities. Nevertheless, our correlative human tissue data, together with our mechanistic *in vitro* cellular data with GrK and endothelial cells, support a model in which extracellular GrK can dampen angiogenesis and thereby reduces tumor growth.

As for translational potential of the above, the GrK-anti-angiogenesis axis could be further explored for the development of new drugs to treat cancer. Irrespective of the location and source of GrK *in vivo*, GrK protein could potentially be therapeutically administered in tumors to trigger sVEGFR1 release from endothelial cells to impair angiogenesis and tumor growth. VEGF-A-inhibitors that prevent angiogenesis and thereby fight cancer, are already widely used in the clinic (34). Moreover, VEGF-A promotes survival of tumor cells *via* different signaling pathways (35) and a recent study demonstrated that sVEGFR1 induces non-apoptotic death in tumor cells (36). If the release of sVEGFR1 by GrK can also be induced *in vivo*, this could have a double or triple anti-tumor effect by inhibiting angiogenesis, and by reducing survival signaling pathways in tumor cells.

## Data Availability Statement

The original contributions presented in the study are included in the article/supplementary material. Further inquiries can be directed to the corresponding author.

## Ethics Statement

The studies involving human participants were reviewed and approved by Biobank review board, University Medical Center Utrecht, Utrecht, The Netherlands. The patients/participants provided their written informed consent to participate in this study.

## Author Contributions

SL: Software, Validation, Formal analysis, Investigation Data, Data Curation, Writing - Review & Editing. CD: Methodology, Investigation. JM: Methodology, Investigation. HK: Methodology, Investigation, Writing - Original Draft. IB: Investigation. AV: Investigation. MK: Investigation. RB: Methodology, Investigation. ML: Methodology, Supervision. RG: Supervision. CC: Supervision. NB: Conceptualization, Writing - Original Draft, Writing - Review & Editing, Supervision. All authors contributed to the article and approved the submitted version.

## Conflict of Interest

The authors declare that the research was conducted in the absence of any commercial or financial relationships that could be construed as a potential conflict of interest.
